# Determining the validity and reliability of spinopelvic parameters through comparing standing whole spinal radiographs and upright computed tomography images

**DOI:** 10.1186/s12891-021-04786-5

**Published:** 2021-10-25

**Authors:** Naruhito Fujita, Mitsuru Yagi, Kota Watanabe, Masaya Nakamura, Morio Matsumoto, Yoichi Yokoyama, Minoru Yamada, Yoshitake Yamada, Takeo Nagura, Masahiro Jinzaki

**Affiliations:** 1 Department of Orthopedic Surgery, Keio University School of Medicine, 35 Shinanomachi, shinjyuku, Tokyo, 160-8582 Japan; 2 Department of Radiology, Keio University School of Medicine, 35 Shinanomachi, Shinjyuku, Tokyo, 160-8582 Japan

**Keywords:** Upright computed tomography, Standing whole spinal radiograph, Spinopelvic parameters, Adult spinal deformity, Interclass correlation coefficient

## Abstract

**Background:**

Standing whole spinal radiographs are used to evaluate spinal alignment in adult spinal deformity (ASD), yet some studies have reported that pelvic incidence, pelvic tilt, and thoracic kyphosis (TK) intra- and inter-observer reliability is low. This study aimed to evaluate the accuracy of spinopelvic parameters through comparing standing whole spinal radiographs and upright CT images.

**Methods:**

We enrolled 26 patients with ASD. All standing whole spinal posterior/anterior and lateral radiographs and upright whole spinal CT had been obtained in a natural standing position. Two examiners independently measured 13 radiographic parameters. Interclass correlation coefficients (ICCs) were used to analyze measurement intra- and inter-observer reliability. Paired t*-* and Pearson’s correlation tests were used to analyze validity of the standing whole spinal radiographs**.**

**Results:**

ICCs of upright CT were excellent in both intra- and inter-observer reliability. However, intra-observer ICCs for TK2–12, TK1–5, TK2–5, and TK5–12 on standing lateral radiographs were relatively low, as were inter-observer ICCs for TK2–12, TK1–5, TK2–5, and TK5–12. Concerning TK values, the difference between the radiographs and CT in TK1–12 and TK2–12 were 4.4 ± 3.1 and 6.6 ± 4.6, respectively, and TK values from T2 showed greater measurement error (*p* < 0.05).

**Conclusions:**

Upright CT showed excellent intra- and inter-observer reliability in the measurement of spinopelvic parameters. Measurement of TK with T2 on standing whole spinal radiographs resulted in a greater measurement error of up to 6.6°. Surgeons need to consider this when planning surgery and measuring postoperative TK changes in patients with ASD.

**Supplementary Information:**

The online version contains supplementary material available at 10.1186/s12891-021-04786-5.

## Background

Sagittal and coronal spinal mal-alignment have been shown to be associated with back pain and disability [1–3]. Glassman et al. reported that positive sagittal mal-alignment was significantly correlated with reduced activities of daily living and quality of life [4]. Schwab et al. proposed a severity classification of adult spinal deformity (ASD) based on coronal and sagittal spinal alignment and reported that C7 sagittal vertical axis (SVA), pelvic tilt (PT), and pelvic incidence (PI) minus lumbar lordosis (LL) mismatch are important indicators when assessing ASD severity [5]. In 2013, Schwab et al. reported that PT > 22 degrees, C7 SVA > 47 mm, and PI-LL > 11 degrees were low health-related quality of life thresholds (HRQoL) [6]. Accurate spinal alignment measurements are therefore important in assessing a patient’s HRQoL. Given spinal alignment varies with weight bearing, it is standard practice currently to measure spinal alignment using standing whole spinal radiographs. However, several potential issues concerning these images have been raised, such as distortion at the edge of the images because the radiograph involves fan beam imaging. Chen et al. reported decreased inter-rater reliability concerning PI and PT compared with whole spinal radiographs taken in a lateral pelvic view involving patients with ASD [7]. Furthermore, upper thoracic vertebrae are difficult to visualize due to overlapping of the rib cage and humerus, and lower thoracic vertebrae also present challenges due to rotation and scoliosis that can result in measurement error; therefore, thoracic kyphosis (TK) in patients with scoliosis is less reliable than the Cobb angle [8, 9].

Computed tomography (CT) has been recognized as the most accurate imaging procedure to determine vertebral shape; however, its use in evaluating spinal posture has been limited since CT scans are undertaken in a supine position. Recent studies have described dynamic changes in spinopelvic parameters between standing and supine positions, including both sagittal and coronal alignment [10, 11]. Therefore, it has been widely recognized that CT images taken in a supine position do not reproduce spinal alignment in the standing position despite the accurate 3-dimensional (3D) reconstruction capability. We recently developed an upright CT with a 320-row multidetector [12], in which a CT scan in a natural standing position can be undertaken.

This study aimed to evaluate the accuracy and reliability of spinopelvic parameters obtained from standing whole spinal radiographic images and upright CT images.

## Methods

The present study was approved by the Japanese Ministry of Health, Labor, and Welfare, Japan Registry of Clinical Trails (jRCTs032180266), and signed informed consent was obtained from all patients.

### Study patients

Twenty-six patients with ASD who met Scoliosis Research Society-Schwab inclusion criteria (major curve Cobb angle, > 30°; or C7 SVA, > 5 cm) [5] and who had undergone posterior/anterior (P/A) and lateral standing whole spinal radiographs and upright CT scans at our institution from April 2019 to June 2020 were prospectively included in this study (men, *n* = 3 [12%]; women, *n* = 23 [88%]; mean age, 64.8 ± 10.0 years; body mass index (BMI), 21.4 ± 3.5 kg/m^2^). Standing P/A and lateral whole spinal radiographs showed that most patients had severe spinal deformity (mean Cobb angle of major curve, 43.4 ± 21.8°; C7 SVA, 90.3 ± 81.1 mm; PI – LL, 34.1 ± 24.4°; PT, 28.9 ± 15.0°). (see Additional file 1, which demonstrates the SRS-Schwab adult spinal deformity classification of all patients.)

### Image acquisition

**Fig. 1:**
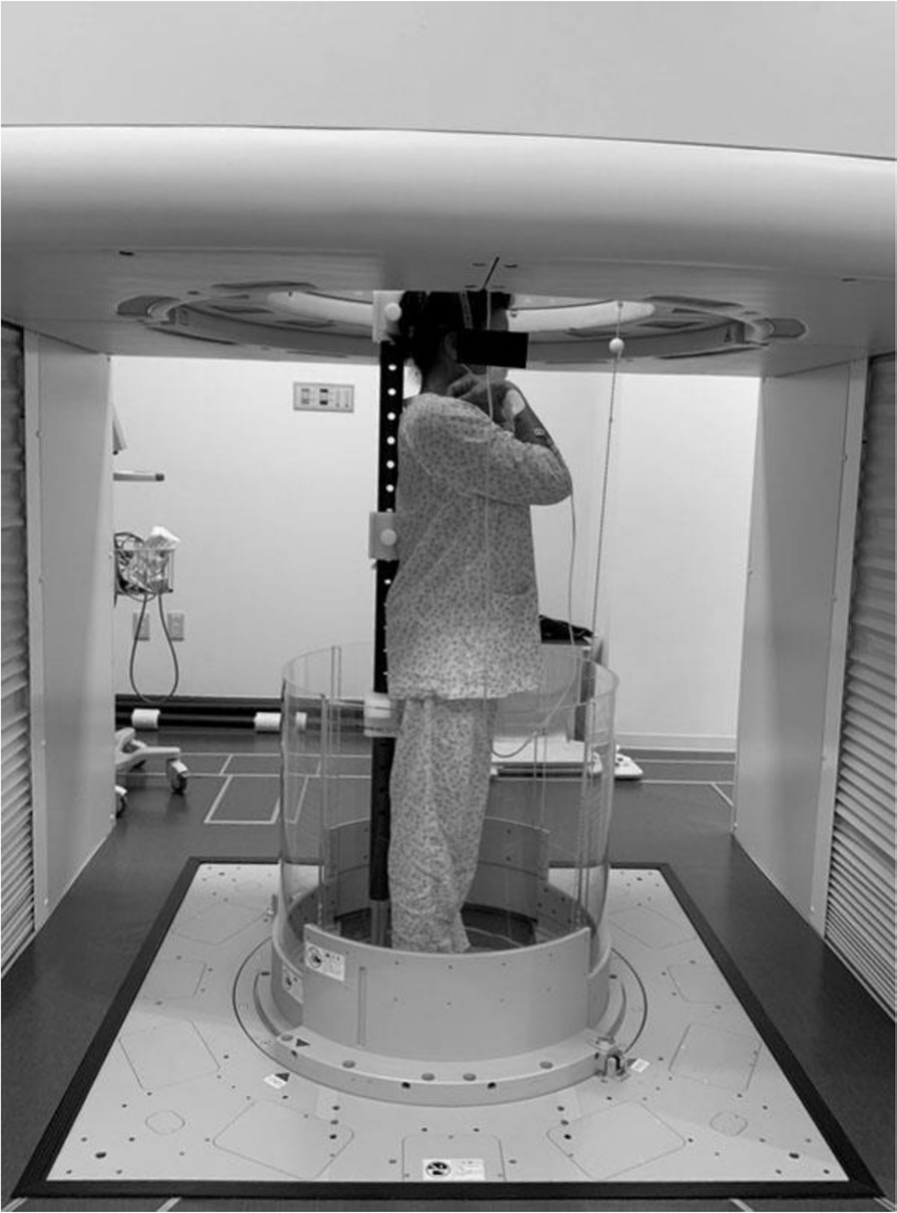
The 320-row upright CT scanner . (Prototype TSX-401R; Canon Medical Systems, Otawara, Japan). During CT scan, the patient’s back was partly touched by the pole for patient safety

All study patients underwent standing whole spinal P/A and lateral radiographs and upright whole spinal CT scans in a natural standing position. Patients were asked to place their fists on their clavicles and to look straight ahead in a relaxed position, while standing barefoot. For lateral radiographs, we standardized the patient’s upper limb position as a fists-on-clavicles position with elbows touching the trunk [13–15]. For patient safety during the examination, patients were asked to make slight contact with a pole positioned behind them during the upright CT scan (Fig. 1).

Radiographs were obtained using the Canon Medical Systems MRAD-A80s (Otawara, Japan). For P/A radiographs, radiography was performed at 85 kV and 320 mA for 0.025 s. For lateral radiographs, radiography was performed at 95 kV, 320 mA for 0.08 s at a focus-film distance of 2.5 m. All radiographic data were saved as Digital Imaging and Communications in Medicine (DICOM) files and exported to PACS (Picture Archiving and Communication System) for analysis.

The CT images were acquired from the level of the external acoustic meatus to the femur including the bilateral femur head using a 320-row upright CT scanner (prototype TSX-401R; Canon Medical Systems, Otawara, Japan) [12, 16, 17]. CT examinations were performed using the following parameters: peak tube voltage, 100 kV; tube current, 10 to 350 mA (using a noise index of 24 for a slice thickness of 5 mm); rotation speed, 0.5 s; and slice thickness, 0.5 mm. Image reconstruction was performed using Adaptive Iterative Dose Reduction 3D (Canon Medical Systems, Otawara, Japan), which reduced the radiation dose [18] . The effective dose of upright CT for the whole spine was approximately 4.9 mSv in this study, which was less than that of a single clinical routine chest CT (7 mSv) described in the literature [19].

The CT data were also accumulated using the DICOM data format, and image data analysis was performed with commercial software (Zed View 14.0.0; LEXI Co., Ltd. Tokyo, Japan). All CT images taken in this study are interpreted by a radiologist and treated individually for patients with abnormal findings.

### Examiners and radiographic measurements

Two board-certified orthopedic surgeons independently measured all radiographic parameters. One examiner measured once and the other examiner measured twice, 1 week apart, following a brief training session on standardizing the measurement. The examiners were blinded to patient clinical information and other measurements. In terms of spinopelvic alignment measurement, we measured the following 13 spinopelvic parameters in this study: Cobb angle of major curve (Cobb), T1 pelvic angle (TPA), cervical lordosis (CL), T1 slope, and TK (TK1–12, TK2–12, TK1–5, TK2–5, TK5–12), LL, PT, PI, and sacral slope (SS). TK was defined as the angle between the superior endplate of the uppermost vertebra and the lower endplate of the lowermost vertebra. LL was defined as the angle from the upper endplate of L1 to the sacral endplate. We referred to the Radiographic Measurement Manual by the Spinal Deformity Study Group [20] for identification and labeling of individual vertebrae and the PI-LL value for each patient was calculated from the measurements. (see Additional file 2, which demonstrates the details of spinopelvic parameters measurement methods.)

### The reference point on upright CT

**Fig. 2:**
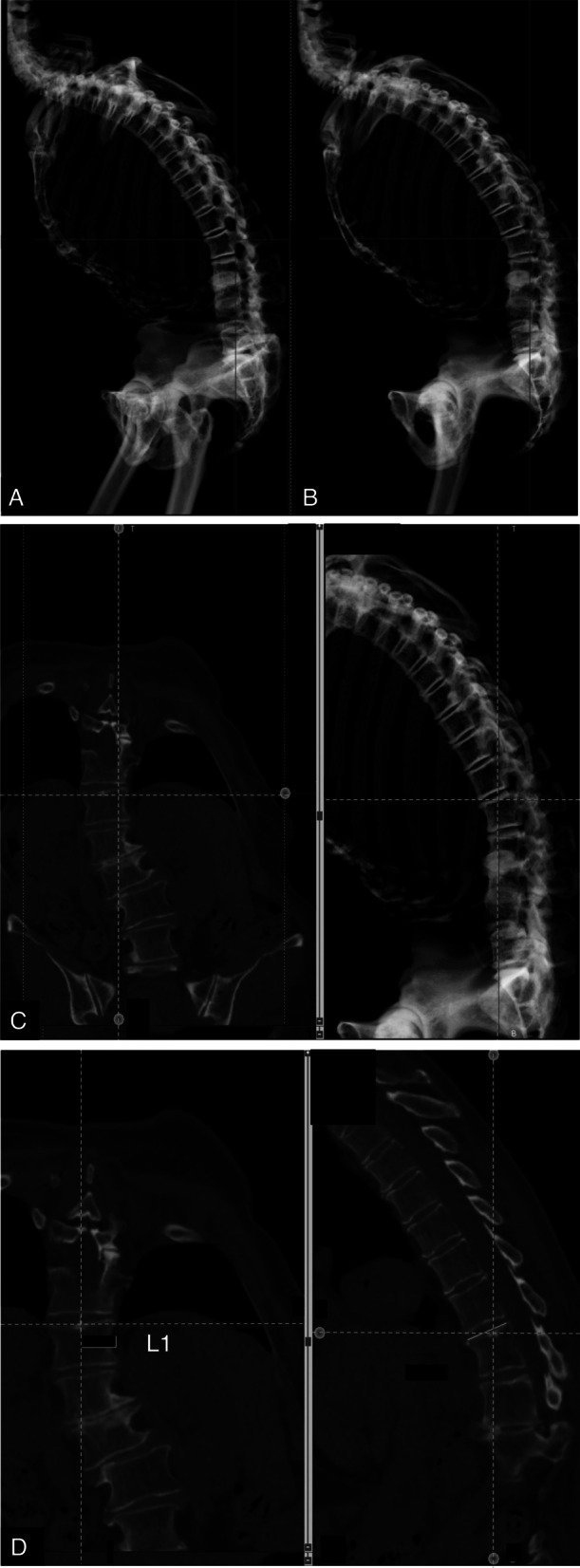
Example of measuring the tilt of sagittal plane of L1 upper endplate. **A**,**B** Rotate axially in DRR image from A to B, and overlay bilateral femoral head. **C** Left: CT image. Right: DRR image. Select the center of the target endplate in the DRR image and obtain the coronal image on that line. (Reference lines are linked between DRR image and CT image.). **D** left: Select the center of the left and right vertebral bodies with the coronal image and configure the sagittal plane. Right: Sagittal CT image use for measurement and L1 upper endplate tilt. (Reference lines are linked each CT images)

In this study, data from upright CT images taken in 3D images were converted to 2-dimensional (2D) images for comparison with values obtained from conventional standing whole spinal radiographs. First, a digitally reconstructed radiograph (DRR) technique was utilized to place the reference point in the converted 2D images acquired from the upright CT images. In DRR, a virtual radiograph light source is set at an arbitrary position in space, parallel lines are drawn from the CT data, and the brightness values ​​on the lines are added to obtain a radiograph-like image with no influence of diffusion. To measure sagittal parameters, the bilateral femoral heads were superimposed to create a true sagittal plane of the spine on a DRR sagittal image. Second, a CT coronal slice perpendicular to the sagittal image was created through cutting the center of the target endplate on a DRR sagittal image. Next, the CT sagittal image, which was obtained through cutting perpendicular to the center of the left and right endplates with the CT coronal slice, was created and measured (Fig. 2). When measuring the coronal parameters, a DRR coronal image was created through accurately rotating the DRR lateral images with the bilateral femoral heads aligned horizontally by 90° to obtain a true coronal DRR image of the spine. A CT sagittal slice was then drawn with a DRR coronal view through cutting it perpendicular to the left and right center of the target endplate. The final CT coronal image was created through cutting perpendicular to the anterior-posterior center of the target endplate on a CT sagittal slice.

### Statistical analyses

The intraclass correlation coefficients (ICCs) of the intra- and inter-observer reliabilities of measurements obtained from standing P/A and lateral whole spinal radiographs and reconstructed upright CT images were calculated. We classified the ICC values according to Aubin et al. criteria as follows: < 0.24 (poor), 0.25–0.49 (low), 0.50–0.69 (fair-to-moderate), 0.70–0.89 (good), and 0.90–1.0 (good-to-excellent) [21].

The relationships between radiographic and CT measurements were compared using correlation analysis (Pearson’s correlation coefficient) and paired t-tests for all parameters. A paired t-test was performed to detect the difference between the radiographic value (RdV) and the CT value (CtV) among the 13 spinopelvic parameters.

Values are expressed as mean ± standard deviation. All statistical analyses were performed using SPSS version 25.0 (IBM Corp., Armonk, NY). The level of significance was set at *p* < 0.05.

## Results

### Intra-observer reliabilities of spinopelvic parameters measured using standing whole spinal radiographs and upright CT scans

The measurements of all radiographic and CT parameters are listed in Tables 1 and 2. Table 3 shows intra-observer reliabilities for 13 spinopelvic parameter measurements of both imaging modalities. The intra-observer ICCs of all parameters measured using upright CT were > 0.9 (0.976–0.997), indicating good-to-excellent reliability. The intra-observer ICCs for TK2–12 (0.841), TK1–5 (0.860), TK2–5 (0.709), and TK5–12 (0.885) on standing lateral radiographs were relatively low, in contrast to the other parameters, indicating good-to-excellent reliability.

**Table 1:** Spinal alignment parameters in standing position measured by radiograph

	Cobb	TPA	CL	T1 slope	TK1–12	TK2–12	TK1–5	TK2–5	TK5–12	LL	PT	PI	SS
**Researcher 1 1st**	43.3 ± 21.8	30.5 ± 18.8	15.3 ± 18.6	28.1 ± 18.9	26.0 ± 19.6	25.0 ± 17.8	8.9 ± 8.3	8.9 ± 5.9	18.5 ± 16.1	18.6 ± 22.9	28.9 ± 15.0	52.8 ± 9.7	23.7 ± 14.5
**Researcher 1 2nd**	42.6 ± 20.5	34 ± 21.8	15.5 ± 20.6	30.2 ± 22.0	26.5 ± 20.3	28.2 ± 19.8	9.5 ± 8.5	7.9 ± 6.1	17.3 ± 15.5	17.7 ± 26.2	28.5 ± 14.6	52.0 ± 9.7	23.9 ± 13.5
**Researcher 2**	41.4 ± 18.2	32.7 ± 19	20.8 ± 21.5	29.3 ± 15.9	25.9 ± 20.3	26.4 ± 19.7	7.3 ± 6.4	7.0 ± 4.6	19.7 ± 16.5	20.7 ± 28.3	29.7 ± 14.6	52.7 ± 11.0	23.1 ± 14.5

Values are shown as mean ± standard deviation.

Units are shown in (°).

**Table 2:** Spinal alignment parameters in standing position measured by CT

	Cobb	TPA	CL	T1 slope	TK1–12	TK2–12	TK1–5	TK2–5	TK5–12	LL	PT	PI	SS
**Researcher 1 1st**	42.5 ± 20.7	31.7 ± 20.1	14.3 ± 21.6	29.7 ± 21.1	27.0 ± 22.0	28.0 ± 21.1	9.2 ± 8.8	10.8 ± 7.2	21.3 ± 19.5	18.5 ± 25.8	30.2 ± 14.4	51.8 ± 10.8	21.4 ± 15.2
**Researcher 1 2nd**	42.7 ± 20.9	32.3 ± 20.5	14.0 ± 20.4	30.7 ± 21.7	26.9 ± 22.5	28.5 ± 22.2	9.6 ± 8.5	11.2 ± 7.6	21.4 ± 19.7	18.6 ± 26.2	30.3 ± 14.6	51.9 ± 11.3	21.5 ± 15.5
**Researcher 2**	43.2 ± 19.3	33.7 ± 20.7	15.2 ± 19.0	30.2 ± 20.5	26.8 ± 22.3	26.1 ± 20.7	9.0 ± 8.5	9.5 ± 6.3	21.8 ± 19.0	19.9 ± 26.3	32.3 ± 15.3	54.5 ± 11.7	22.7 ± 15.9

Values are shown as mean ± standard deviation.

Units are shown in (°).

**Table 3:** Intra- and Inter-rater reliability ICC values

**Intra-rater reliability**	**Cobb**	**TPA**	**CL**	**T1 slope**	**TK1–12**	**TK2–12**	**TK1–5**	**TK2–5**	**TK5–12**	**LL**	**PT**	**PI**	**SS**
**Xp**	0.986(0.970–0.994)	0.938(0.868–0.972)	0.938(0.868–0.972)	0.962(0.918–0.983)	0.97(.0935–0.986)	0.841(0.680–0.925)	0.86(0.715–0.934)	0.709(0.453–0.857)	0.885(0.763–0.947)	0.924(0.840–0.965)	0.932(0.856–0.969)	0.912(0.815–0.959)	0.916(0.824–0.961)
	*p* < 0.001	*p* < 0.001	*p* < 0.001	*p* < 0.001	*p* < 0.001	*p* < 0.001	*p* < 0.001	*p* < 0.001	*p* < 0.001	*p* < 0.001	*p* < 0.001	*p* < 0.001	*p* < 0.001
**CT**	0.996(0.990–0.998)	0.994(0.987–0.997)	0.990(0.978–0.995)	0.995(0.988–0.998)	0.996(0.992–0.998)	0.995(0.990–0.998)	0.983(0.963–0.992)	0.976(0.949–0.989)	0.993(0.985–0.997)	0.997(0.993–0.999)	0.997(0.994–0.999)	0.98(0.956–0.991)	0.994(0.988–0.997)
	*p* < 0.001	*p* < 0.001	*p* < 0.001	*p* < 0.001	*p* < 0.001	*p* < 0.001	*p* < 0.001	*p* < 0.001	*p* < 0.001	*p* < 0.001	*p* < 0.001	*p* < 0.001	*p* < 0.001
**Inter-rater reliability**	**Cobb**	**TPA**	**CL**	**T1 slope**	**TK1–12**	**TK2–12**	**TK1–5**	**TK2–5**	**TK5–12**	**LL**	**PT**	**PI**	**SS**
**Xp**	0.891(0.774–0.949)	0.908(0.806–0.957)	0.912(0.633–0.969)	0.924(0.840–0.965)	0.919(0.827–0.963)	0.770(0.550–0.890)	0.784(0.575–0.897)	0.637(0.333–0.820)	0.791(0.587–0.900)	0.883(0.759–0.946)	0.883(0.756–0.946)	0.804(0.609–0.907)	0.866(0.724–0.938)
	*p* < 0.001	*p* < 0.001	*p* < 0.001	*p* < 0.001	*p* < 0.001	*p* < 0.001	*p* < 0.001	*p* < 0.001	*p* < 0.001	*p* < 0.001	*p* < 0.001	*p* < 0.001	*p* < 0.001
**CT**	0.978(0.953–0.990)	0.992(0.838–0.998)	0.938(0.868–0.972)	0.981(0.958–0.991)	0.971(0.935–0.987)	0.980(0.947–0.992)	0.929(0.848–0.967)	0.929(0.809–0.971)	0.988(0.973–0.994)	0.982(0.961–0.992)	0.982(0.839–0.995)	0.931(0.681–0.977)	0.986(0.955–0.994)
	*p* < 0.001	*p* < 0.001	*p* < 0.001	*p* < 0.001	*p* < 0.001	*p* < 0.001	*p* < 0.001	*p* < 0.001	*p* < 0.001	*p* < 0.001	*p* < 0.001	*p* < 0.001	*p* < 0.001

Values are shouwn as ICC(95%confidence interval)

### Inter-observer reliabilities of spinopelvic parameters measured using standing whole spinal radiographs and upright CT scans

Table 3 shows inter-observer reliabilities for 13 spinopelvic parameter measurements of both imaging modalities. Inter-observer ICCs of all parameters measured using upright CT scans were > 0.9 (0.929–0.992), indicating good-to-excellent reliability. The inter-observer ICCs of radiographs for TK2–12 (0.770), TK1–5 (0.784), TK2–5 (0.637), and TK5–12 (0.791) were < 0.8, with TK2–5 showing the lowest reliability, and categorized as fair-to-moderate. These results indicated that TK parameters including T2 or T5 in radiographic measurements tended to worsen ICC values.

### The difference between radiograph and CT values for spinopelvic parameters

**Fig. 3:**
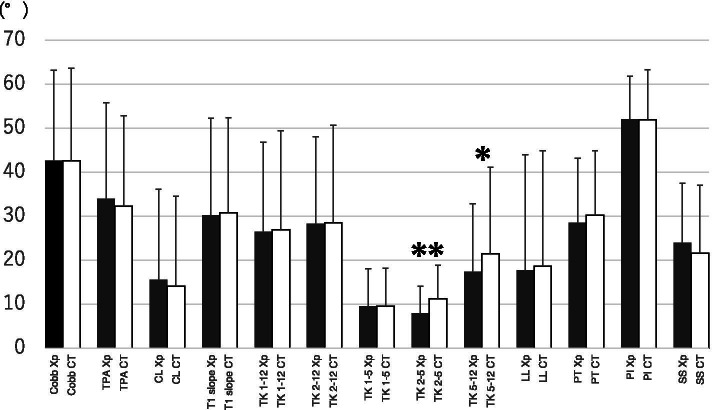
Mean (+ standard deviation) values of spinopelvic parameters measured by radiograph (black bars) and CT (white bars). Asterisk exhibit statistically difference (*p* < 0.05), double asterisks exhibit statistically difference (*p* < 0.01)

Table 4 shows the absolute difference between RdV and CtV for each parameter. The largest difference was found in CL (9.2 ± 6.4°), followed by TK2–12 (6.6 ± 4.6°) and LL (6.6 ± 8.5°). There was a statistically significant difference between RdV and CtV in TK2–5 (*p* = 0.008) and TK5–12 (*p* = 0.022). However, there was no statistically significant difference between RdV and CtV in the lumbopelvic parameters (Table 5, Fig. 3). Pearson’s correlation coefficients between RdV and CtV for each parameter are listed in Table 5. The correlation coefficients between RdV and CtV of other parameters were > 0.8 (0.831–0.972), except for the relatively inferior positive correlation in TK2–5 (*r* = 0.659).

**Table 4:** The difference between radiograph and CT values

Cobb	TPA	CL	T1 slope	TK1–12	TK2–12	T1–5	TK2–5	TK5–12	LL	PT	PI	SS
4.2 ± 2.5	6.4 ± 5.4	9.2 ± 6.4	4.5 ± 3.0	4.4 ± 3.1	6.6 ± 4.6	4.1 ± 2.6	5.0 ± 4.4	6.4 ± 6.3	6.6 ± 8.5	5.2 ± 3.7	3.8 ± 3.5	6.3 ± 5.2

Values are shown as mean ± standard deviation

Units are shown in (°)

**Table 5:** The agreement of the spinopelvic parameters on the radiographs and CT values

	Cobb	TPA	CL	T1 slope	TK1–12	TK2–12	TK1–5	TK2–5	TK5–12	LL	PT	PI	SS
***p*-value**	0.951	0.182	0.506	0.585	0.666	0.849	0.910	0.008	0.022	0.687	0.253	0.924	0.141
**Correlation Coefficient**	0.971	0.923	0.850	0.968	0.972	0.930	0.831	0.659	0.923	0.914	0.909	0.883	0.859

An additional analysis was performed to test whether thoracic measurement from T1 had less accuracy than that from T2. The differences in RdV and CtV between TK1–12 and TK2–12 were 4.4 ± 3.1° and 6.6 ± 4.6°, respectively, and this difference was statistically significant (*p* < 0.05). The differences in RdV and CtV between TK1–5 and TK2–5 were 4.1 ± 2.6° and 5.0 ± 4.4°, respectively, and no significant difference was observed (*p* = 0.760). (see Additional file 3, which shows t-test results as error bar graphs.)

## Discussion

Although coronal and sagittal standing whole spinal radiographs have been the gold standard for measuring spinal alignment since 1950, analysis using the slot-scanning system (EOS® imaging system, Paris, France) has become more popular in recent years. The EOS® imaging system is a low-dose biplanar digital radiographic imaging system that has two linear X-ray sources and two gaseous detector arrays that move together to scan the patient, enabling 3D semi-automatic measurement as well as 2D manual measurement [22]. In semi-automatic measurement, when the 3D vertebral model is manually adjusted and overlaid on the X-ray image, the measurement is automatically performed by sterEOS 3D workstation software. Somoskeoy et al. reported ICCs between manual and automatic measurements using the EOS® imaging system for spinopelvic parameters measurement of 201 individuals. They concluded that intra-observer reliability of manual measurement and semi-automatic measurement was 0.994–1.000, 0.993–1.000, respectively, and inter-observer reliability was 0.844–0.971 and 0.930–0.985, respectively [23]. In this study, inter-observer reliability of upright CT parameters ranged from 0.976 to 0.997 and inter-observer reliability ranged from 0.929 to 0.992. The findings of the present study indicated that the ICCs of the spinopelvic parameters obtained from upright CT were equivalent to those obtained in the previously described EOS® imaging system using semi-automatic measurement. However, because semi-automatic measurement in the EOS® imaging system is based on virtual 3D images reconstructed using a model bone from a plain radiograph taken simultaneously in two directions, discordance between the real spine shape and the reconstructed images occurs, especially in patients with marked degenerative changes and osteophyte formation of the vertebra such as occurs in patients with ASD [24]. The application of an upright CT enabled us to measure the whole spinal curvature from the patient’s original vertebral body, which helped to overcome the major limitation of EOS.

Among the described radiographic parameters, the ICCs of the TK, except for TK1–12, were relatively low in terms of intra- and inter-observer reliability, and TK2–5 had the lowest intra- and inter-observer reliability (TK2–5: intra-observer reliability, 0.709; inter-observer reliability, 0.637). The inferior correlation coefficient of TK2–5 between radiographs and upright CT images also supported this finding.

The ICC was > 0.9 in TK1–12 measurement; therefore, it can be concluded that the accuracy of the TK measurement in standing whole spinal radiographs may decrease when T2 and T5 are used as the end vertebrae for each measurement. This tendency was also observed in the results of the paired t-test in TK2–5 and TK5–12 in our study. Statistically significant differences were observed in T2–5 and T5–12 among all parameters. Our study findings follow and extend those of previous studies. Kuklo et al. described the accuracy of radiographic measurements in 30 patients with adolescent idiopathic scoliosis and concluded that T2–5 kyphosis had the lowest intra- and inter-rater reliability among various TK measurements [25].

This low TK measurement reliability in the standing lateral radiographs was mainly because of poor visualization of vertebral endplates due to structural overlap of the humeral head and upper thoracic rib cage in the upper thoracic spine and lateral and rotational deformity of the curvature in the lower thoracic spine [8, 9].

In the TK measures, the T1 upper endplate was used as the cranial landmark for TK1–12 measurement, and the T2 upper endplate was used for TK2–12 measurement. However, sometimes the T1 upper endplate was recognizable, but the T2 upper endplate was poorly visualized because of humeral head, shoulder girdle, and rib cage overlap. (see Additional file 4, which shows a representative case example of a cervical thoracic junction in a standing lateral whole spinal radiograph.)

Therefore, to determine whether there was a measurement difference between the measures from T1 and the measures from T2 in the TK on the standing lateral spine radiographs, the differences between the RdV and CtV of TK1–5 and TK2–5, and the RdV and CtV of TK1–12 and TK2–12, were analyzed using t-tests. As expected, the difference between the RdV and CtV of TK 1–12 was significantly less than that of TK2–12. However, no significant difference was found between TK1–5 and TK2–5. Therefore, TK1–12 can be used as a more accurate measure of an individual’s TK with better inter- and intra-observer reliability.

Regarding the correlation coefficient of the RdV and CtV concerning the lumbar spine parameters and lumbopelvic parameters, the correlation coefficients of PI and SS were 0.883 and 0.859, respectively, which were lower than 0.9, and can be considered relatively low.

Among the lumbar spinal and lumbopelvic parameters, inter-observer reliability was also the lowest at PI (0.804), followed by SS (0.866). Yamada et al. reported that intra- and inter-observer reliability for each pelvic parameter measured using standing whole spinal radiographs was PI (0.84 and 0.79), SS (0.87 and 0.83), and PT (0.98 and 0.96), respectively, among 120 patients with spinal disease [26]. There was agreement between previous studies and this study that the inter-observer reliability of PI and SS was low. It has been recognized that there is a large measurement error in PI and SS due to poor visualization and inaccuracy of identification of the sacral endplate. To our knowledge, no study has compared the ICCs of spinopelvic parameters measured using standing whole spinal radiographs of the thoracic and lumbar spine simultaneously. Our study findings indicate that the ICCs of TK were even lower than those of lumbopelvic parameters. Further, significant measurement differences between RdV compared with those of CtV were found in TK but not in lumbopelvic parameters.

This study had several limitations. First, standing whole spinal radiographs and upright CT scans were not taken simultaneously but were taken in a different location within the same building. It is possible that patient postures were not exactly the same for each examination. However, the examiners instructed the patients to assume the same posture during CT scans and radiographs, and our study protocol helped to minimize postural differences between the two examinations. Second, Chen et al. compared the reliability of whole spinal radiographs and pelvic radiographs and reported that pelvic parameter measurement accuracy was higher using pelvic radiography. This was because the X-ray irradiation angle was obliquely irradiated to the pelvis during whole spinal imaging, and errors were more likely to occur compared with a side view of the pelvis obtained when irradiated in a straight line. In our study, we also used whole spinal radiograph images for pelvic parameter measurement to reduce the radiation dose [7].

## Conclusions

In this study, we evaluated the accuracy of standing whole spinal radiographs compared with upright CT scans in spinopelvic parameter measurement. First, upright CT had an excellent ICC for both intra- and inter-observer reliability. Among the spinopelvic parameters, the ICC for TK measures had low intra- and inter-rater reliability. Moreover, TK2–5 and TK5–12 were significantly different in terms of RdV and CtV. TK measurements using standing whole spinal radiographs resulted in large errors, mainly due to the lack of a clear visualization of the endplates of each vertebra due to overlap from the rib cage, humeral head, and deformed vertebral bodies. Therefore, special attention is needed when planning surgery and measuring postoperative TK changes in patients with ASD. This study showed that measurement accuracy was greater in TK1–12 than in T2–12; therefore, we recommend using TK1–12 instead of T2-T12 in clinical use.

## Supplementary Information

**Additional file 1.**

**Additional file 2.**

**Additional file 3.**

**Additional file 4.**

## Abbreviations

ASD: Adult spinal deformity
BMI: Body mass index
CL: Cervical lordosis
Cobb: Cobb angle of major curve
CT: Computed tomography
CtV: CT value
DICOM: Digital Imaging and Communications in Medicine
DRR: Digitally reconstructed radiograph
HRQoL: Health-related quality of life
ICCs: Interclass correlation coefficients
LL: Lumbar lordosis
PACS: Picture archiving and communication system
PI: Pelvic incidence
PT: Pelvic tilt
RdV: Radiographic value
SVA: Sagittal vertical axis
SS: Sacral slope
TK: Thoracic kyphosis
TPA: T1 pelvic angle
2D: 2-dimensional
3D: 3-dimensional.
