# Supplementary figures and images for: Determining the validity and reliability of spinopelvic parameters through comparing standing whole spinal radiographs and upright computed tomography images
Source: BMC Musculoskelet Disord. 2021 Oct 25;22:899. doi: 10.1186/s12891-021-04786-5 (PMC8546937; doi:10.1186/s12891-021-04786-5)

## Slide 1
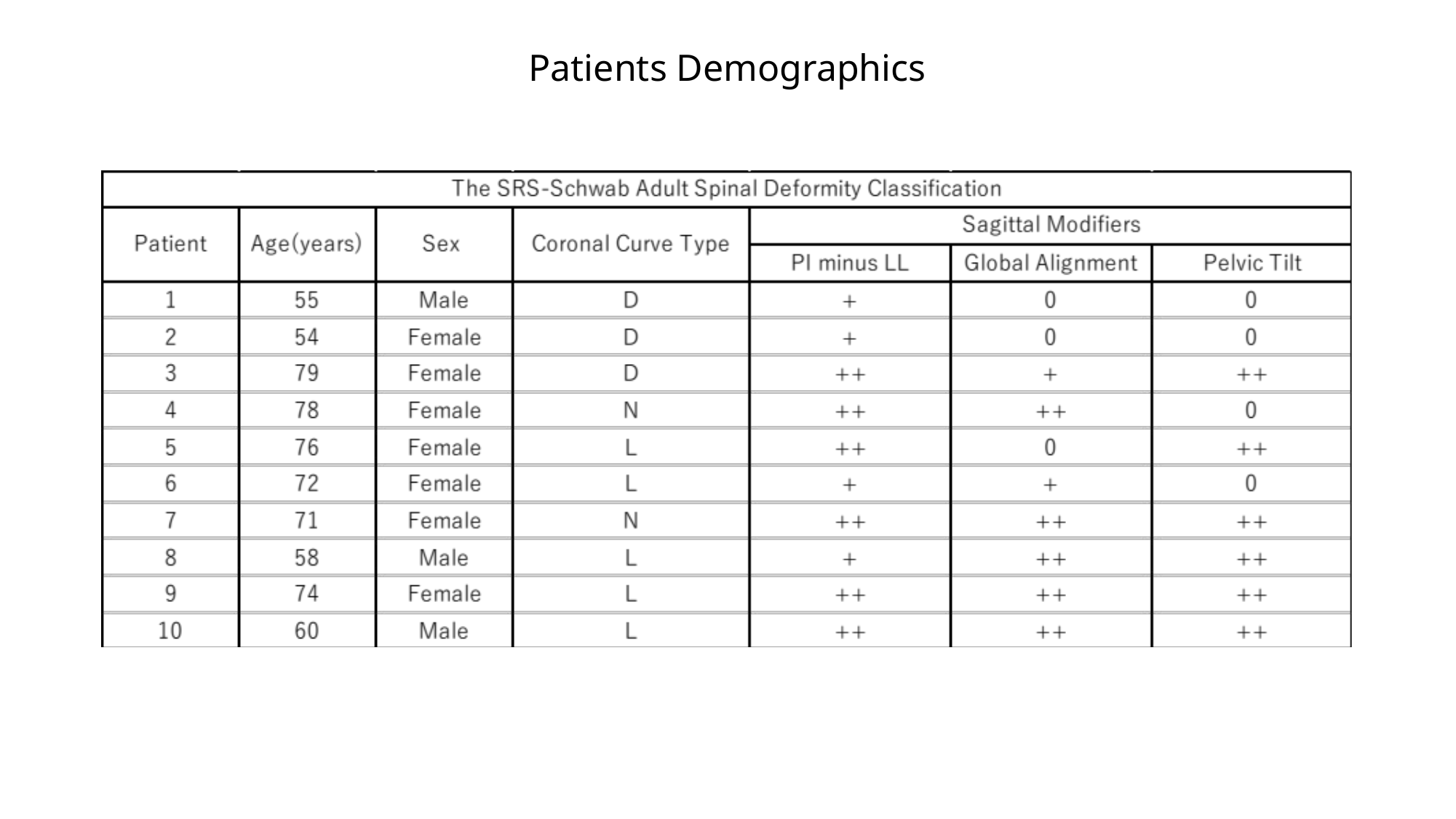

Patients Demographics

## Slide 2
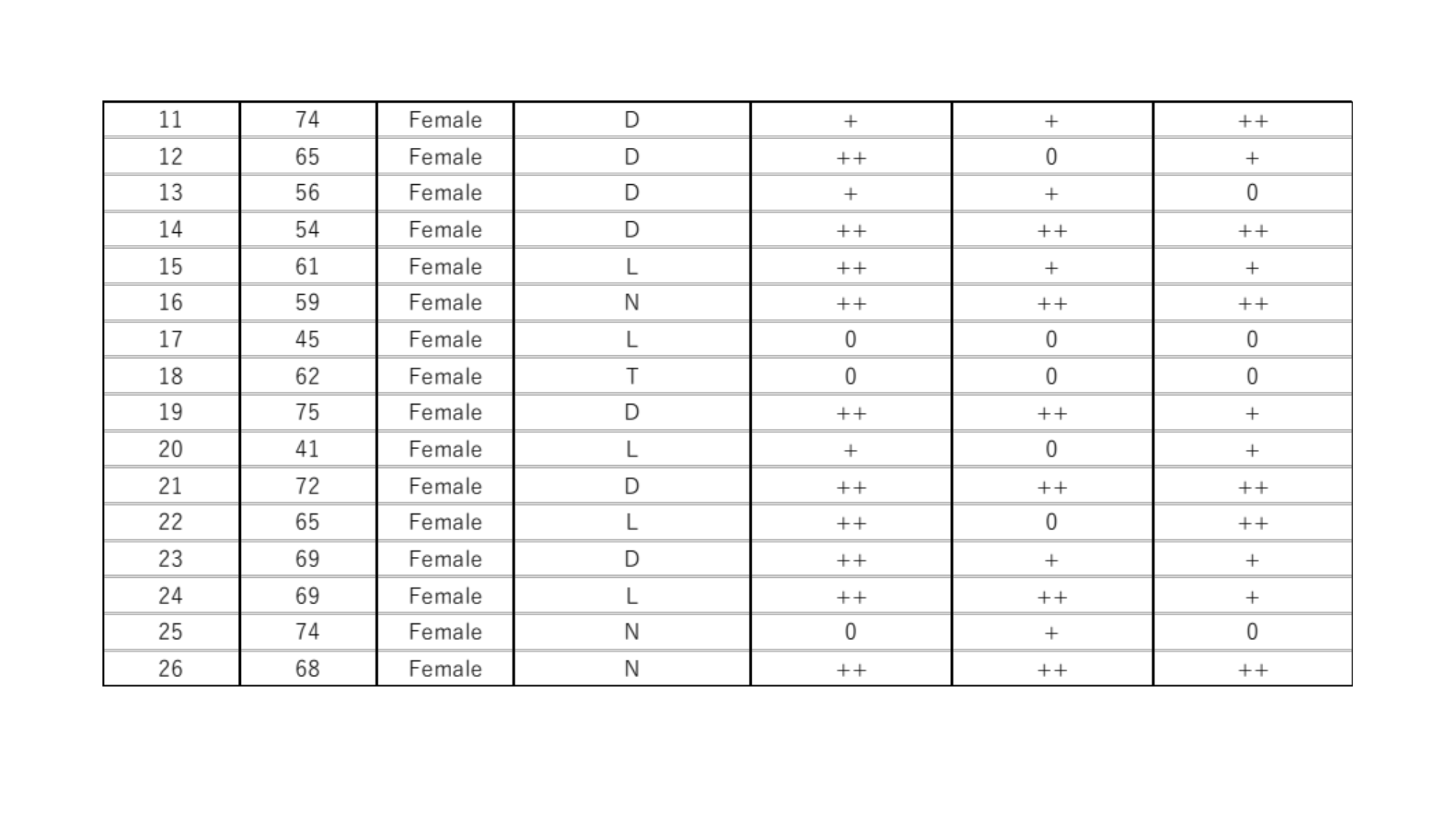

Supplement: Supplementary file 1 — Additional file 1. [file 12891_2021_4786_MOESM1_ESM.pptx]
