# Supplementary material for: Determining the validity and reliability of spinopelvic parameters through comparing standing whole spinal radiographs and upright computed tomography images
Source: BMC Musculoskelet Disord. 2021 Oct 25;22:899. doi: 10.1186/s12891-021-04786-5 (PMC8546937; doi:10.1186/s12891-021-04786-5)
Supplement: Supplementary file 2 — Additional file 2. [file 12891_2021_4786_MOESM2_ESM.pptx]

## Slide 1
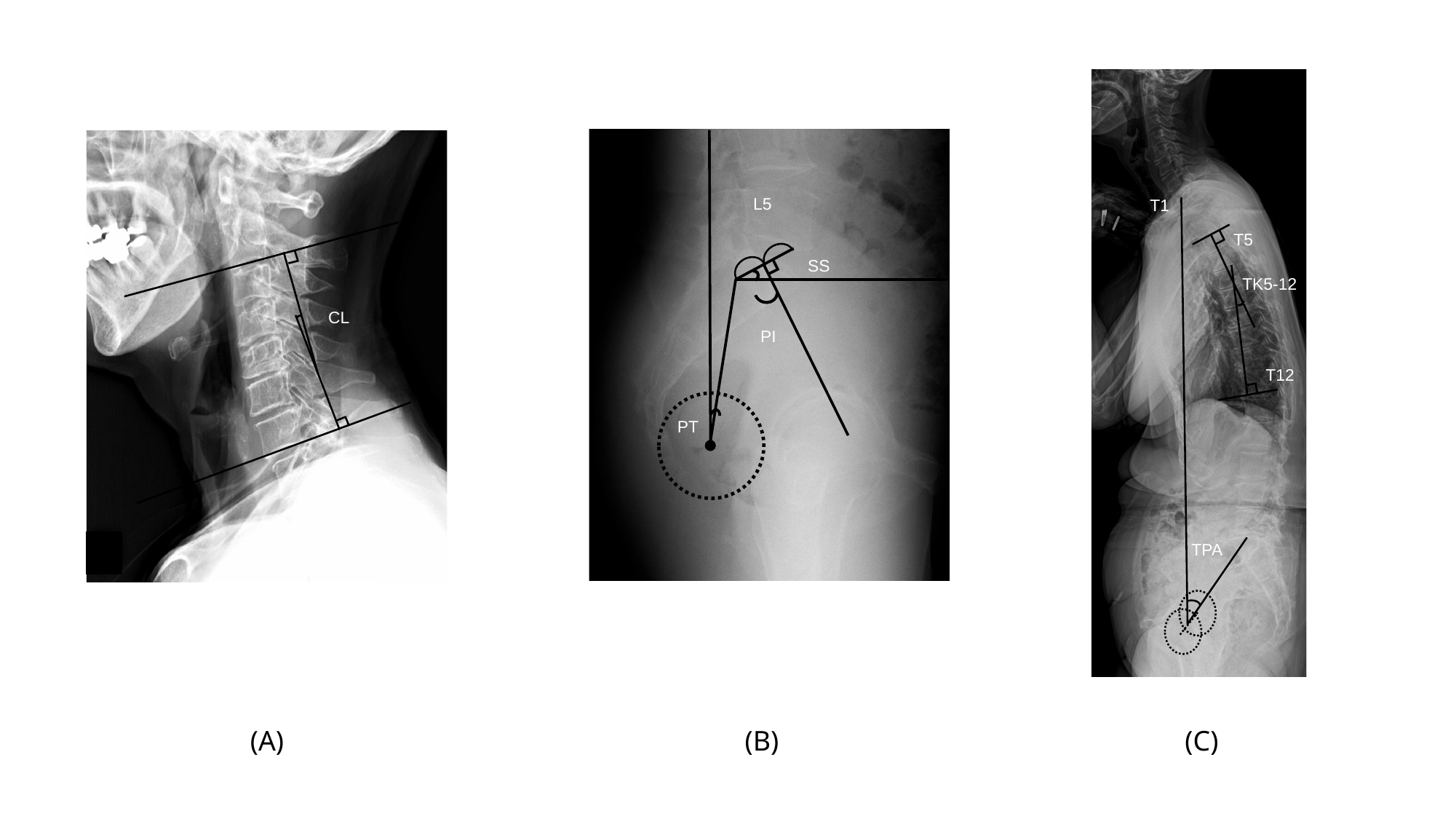

T1
T5
TK5-12
T12
TPA
L5
SS
PI
PT
CL
(A)
(B)
(C)

## Slide 2
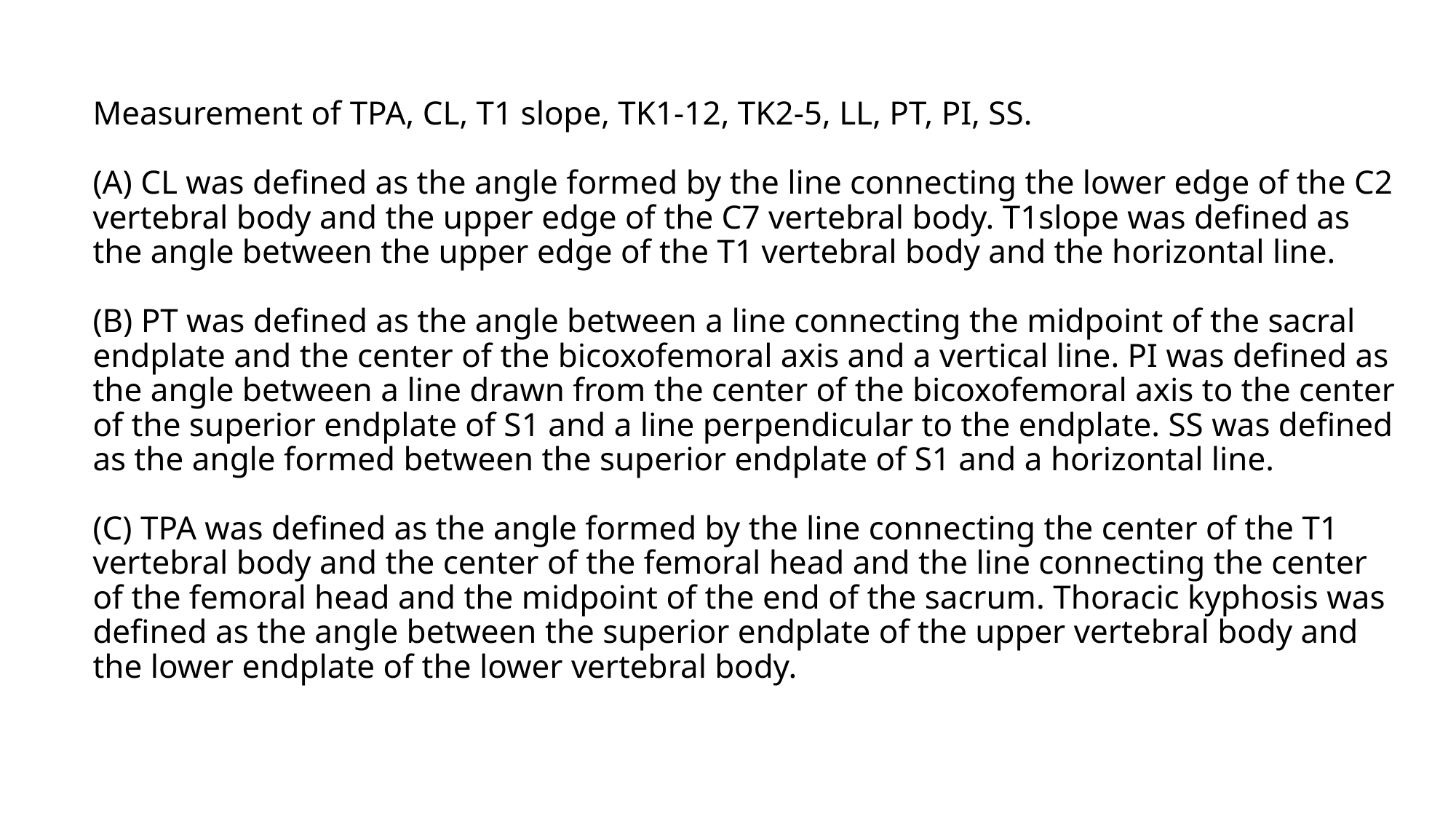

# Measurement of TPA, CL, T1 slope, TK1-12, TK2-5, LL, PT, PI, SS. (A) CL was defined as the angle formed by the line connecting the lower edge of the C2 vertebral body and the upper edge of the C7 vertebral body. T1slope was defined as the angle between the upper edge of the T1 vertebral body and the horizontal line. (B) PT was defined as the angle between a line connecting the midpoint of the sacral endplate and the center of the bicoxofemoral axis and a vertical line. PI was defined as the angle between a line drawn from the center of the bicoxofemoral axis to the center of the superior endplate of S1 and a line perpendicular to the endplate. SS was defined as the angle formed between the superior endplate of S1 and a horizontal line. (C) TPA was defined as the angle formed by the line connecting the center of the T1 vertebral body and the center of the femoral head and the line connecting the center of the femoral head and the midpoint of the end of the sacrum. Thoracic kyphosis was defined as the angle between the superior endplate of the upper vertebral body and the lower endplate of the lower vertebral body.
