# Supplementary material for: Determining the validity and reliability of spinopelvic parameters through comparing standing whole spinal radiographs and upright computed tomography images
Source: BMC Musculoskelet Disord. 2021 Oct 25;22:899. doi: 10.1186/s12891-021-04786-5 (PMC8546937; doi:10.1186/s12891-021-04786-5)
Supplement: Supplementary file 3 — Additional file 3. [file 12891_2021_4786_MOESM3_ESM.pptx]

## Slide 1
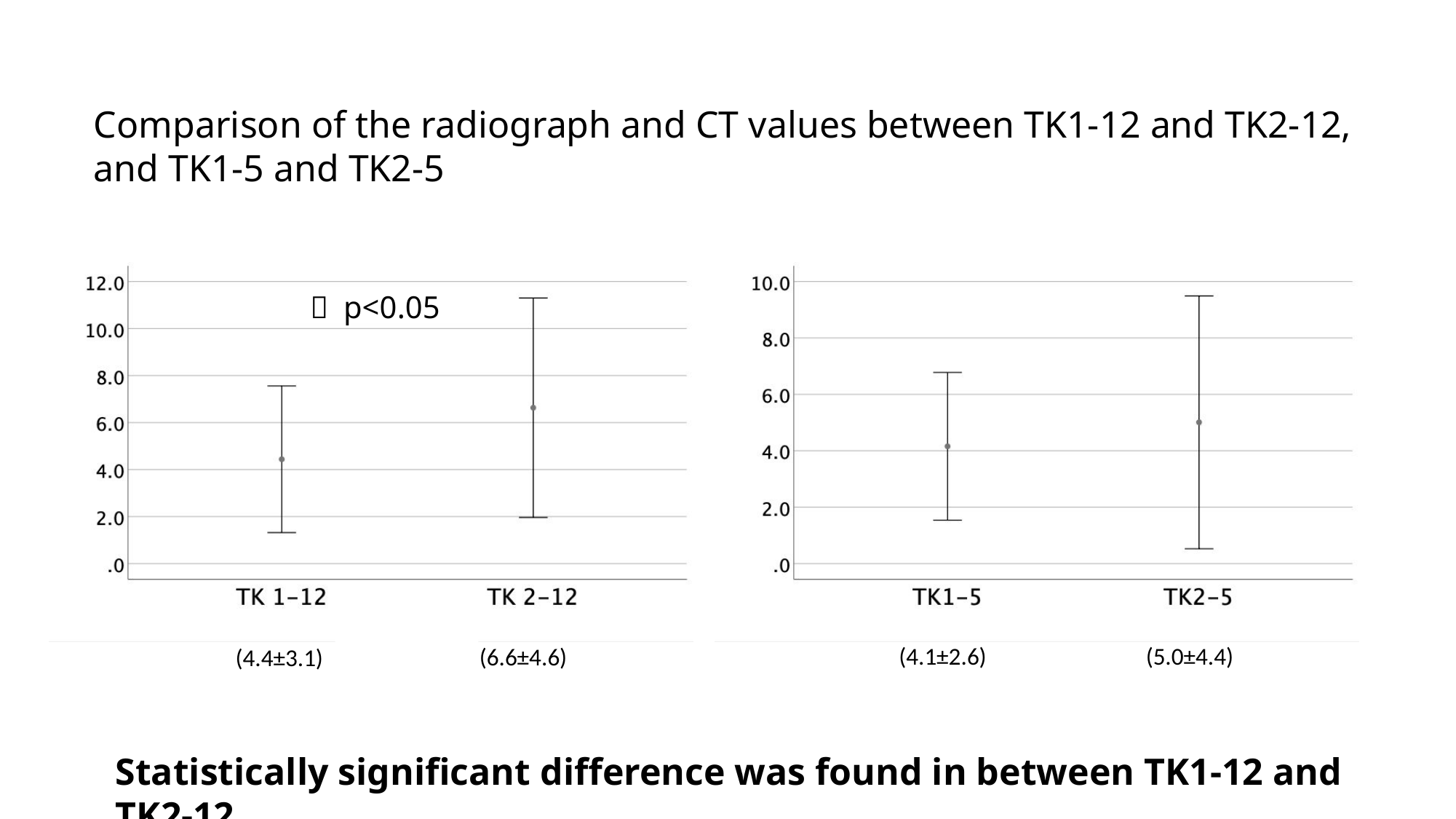

Comparison of the radiograph and CT values between TK1-12 and TK2-12, and TK1-5 and TK2-5
＊ p<0.05
(4.1±2.6)
(5.0±4.4)
(6.6±4.6)
(4.4±3.1)
Statistically significant difference was found in between TK1-12 and TK2-12.
