# Supplementary material for: Determining the validity and reliability of spinopelvic parameters through comparing standing whole spinal radiographs and upright computed tomography images
Source: BMC Musculoskelet Disord. 2021 Oct 25;22:899. doi: 10.1186/s12891-021-04786-5 (PMC8546937; doi:10.1186/s12891-021-04786-5)
Supplement: Supplementary file 4 — Additional file 4. [file 12891_2021_4786_MOESM4_ESM.pptx]

## Slide 1
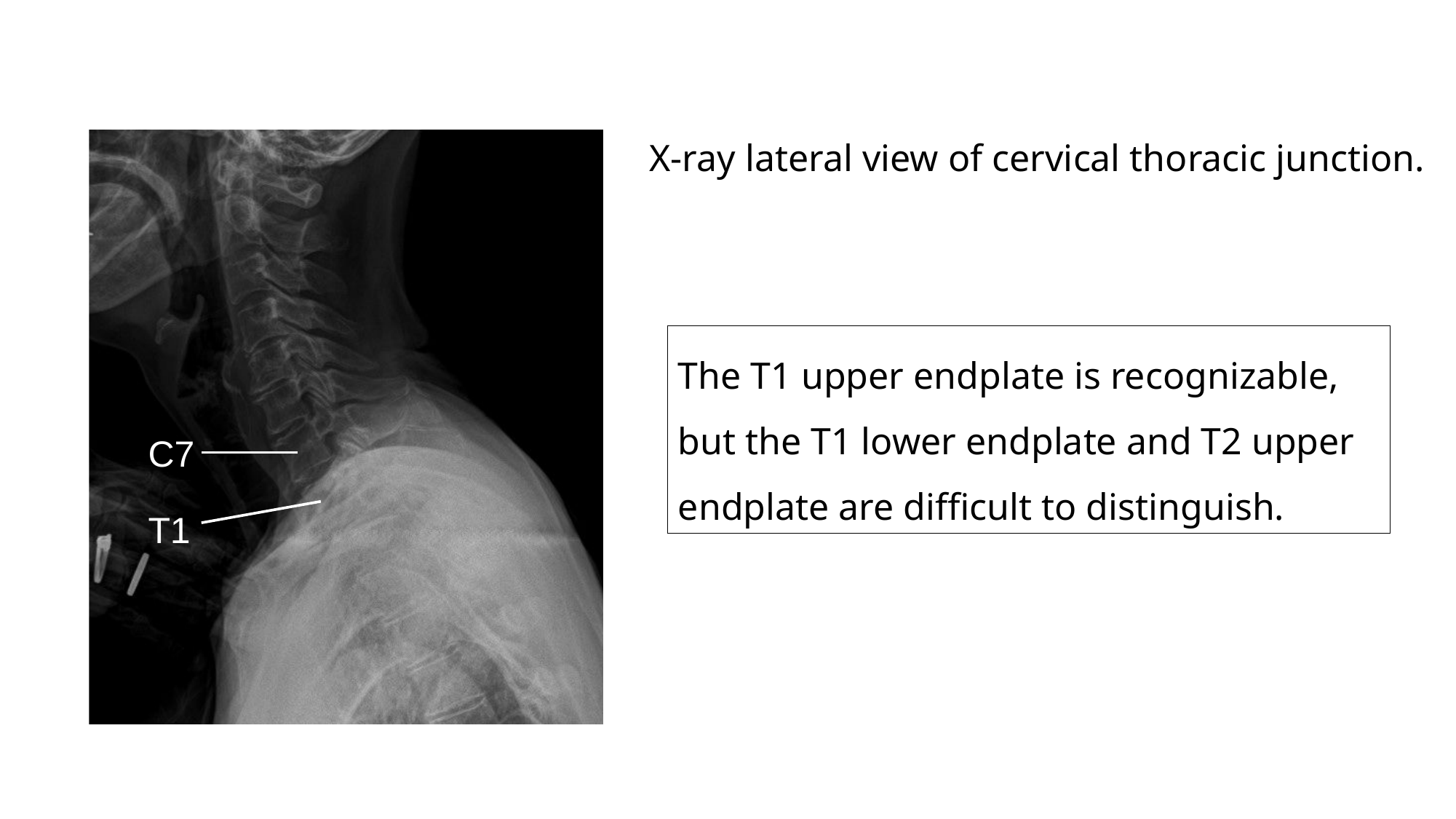

X-ray lateral view of cervical thoracic junction.
The T1 upper endplate is recognizable, but the T1 lower endplate and T2 upper endplate are difficult to distinguish.
C7
T1
